# Wideband Dual-Polarized Octagonal Cavity-Backed Antenna with Low Cross-Polarization and High Aperture Efficiency

**DOI:** 10.3390/s23020731

**Published:** 2023-01-09

**Authors:** Yanxia Liu, Dustin Isleifson, Lotfollah Shafai

**Affiliations:** 1 Department of Electrical and Computer Engineering, University of Manitoba, Winnipeg, MB R3T 5V6, Canada; 2 Centre for Earth Observation Science, Clayton H. Riddell Faculty of Environment, Earth and Resources, University of Manitoba, Winnipeg, MB R3T 2N2, Canada

**Keywords:** cavity-backed antenna, dual-polarization, differential feed, wide bandwidth, low cross-polarization, high aperture efficiency

## Abstract

Simultaneously enhancing multiple antenna performance parameters is a demanding task, especially with a challenging set of design goals. In this paper, by carefully deriving a compatible set of enhancement techniques, we propose a compact/lightweight/low-cost high-performance L-band octagonal cavity-backed hybrid antenna with multiple attractive features: dual-polarization, wide impedance bandwidth, low cross-polarization, high gain, and high aperture efficiency. The ground cavity is octagonal, which allows the antenna to have a small footprint, and, more importantly, low cross-polarization and high aperture efficiencies when compared to a commonly-used square design. The hybrid design relies on the resonance merging of two radiating elements, i.e., radiating feedlines and a conductive open prism, to form a wide impedance bandwidth. To permit polarization diversity and low cross-polarization, it is differentially and orthogonally fed. Herein, a series of parametric simulation studies on antenna configurations provide information on how to improve the impedance bandwidth and cross-polarization performance. To verify the simulation studies, an antenna prototype was fabricated and tested. Excellent agreement between the simulated and measured results was reached.

## 1. Introduction

An antenna, as one of the key components in a wireless device, has a vital impact on device performance. To fulfill the requirements of the application for which the antenna is used, a certain set of antenna performance parameters needs to be met. Simultaneously enhancing multiple antenna performance parameters can be challenging but necessary for many applications. On an individual basis, performance enhancement on single parameters has been studied, and numerous enhancement techniques have been developed. However, it takes specialized methods, such as those discussed in this manuscript, to achieve high performance for multiple parameters while not sacrificing the gains made in the enhancement of individual factors. In this paper, we propose a compact/lightweight/low-cost, high-performance antenna with a challenging combination of qualities: polarization diversity, wide impedance bandwidth, high gain with stable broadside radiation, low cross-polarization, and high aperture efficiency. Such an antenna is desirable for many applications, including remote sensing and satellite communications.

Impedance bandwidth enhancement is often one of the first steps in antenna optimization. Two of the most widely used techniques are parasitic element loading [1,2,3,4] and a hybrid approach [5,6,7,8]. Both methods achieve wide bandwidths by combining resonances caused by multiple radiators (and the coupling between them). For successful merging, these resonances need to be relatively close to each other and have the same type of radiation (e.g., broadside or end-fire). In the case of parasitic element loading, the feeding structure does not radiate. The only radiators are the driven antenna and the parasitic elements. With the hybrid approach, the feeding structure acts as an effective radiator and provides additional resonance(s) [6]. Parasitically loaded antennas gain additional resonance(s) by introducing extra antenna component(s), while hybrid antennas can have a wide bandwidth without any additional component(s). Because of this, hybrid designs can be more compact and simpler than parasitically loaded ones. Radiating slots and monopoles are two of the most common feeding structures for hybrid antennas. Various types of antennas have been integrated with them to make wideband hybrid antennas, e.g., slot/patch antenna [5], slot/dielectric resonator antenna [6], slot/patch/dielectric resonator antenna [7], and monopole/dielectric resonator antenna [8].

Gain enhancement approaches include: forming an array [9], using a ground cavity [10,11,12,13], parasitic element loading [1,2,3,4], and partially reflective surface loading [14,15]. These studies investigate mostly gain enhancement techniques but have not addressed the aperture efficiency of the antennas, which result in a compact geometry and wide overlapping impedance, gain, and cross-polarization bandwidths. Among these techniques, only the use of a ground cavity can simultaneously provide gain enhancement and low cross-polarization. More importantly, a cavity-backed configuration is also known to support wideband operation [13]. Consequently, choosing such a configuration shows potential for simultaneous enhancement of multiple antenna performance parameters.

Existing cross-polarization reduction techniques include feed designing [16,17] and shaping [16,18]. Differential feeding [16] and aperture-coupled feeding [17] are two of the most frequently used feed designs for suppressing cross-polarization. With differential feeding, there is zero cross-polarization in principal planes if the antenna is geometrically symmetric [19]. However, this method is less effective in diagonal planes where maximum cross-polarization occurs [20]. In aperture-coupled feeding, the radiation from feedlines is shielded by ground planes, and as a result, the cross-polarization due to the feedlines is suppressed [21]. The abrupt discontinuity at edges, especially sharp corners, induces non-uniform and asymmetric currents, which cause cross-polarized radiation [16,18]. With appropriate shaping, surface currents can be redistributed in such a way that cross-polarized components are reduced and/or co-polarized components are increased. The majority of these techniques were developed for reducing cross-polarization in principal planes. Therefore, they either are not as effective in diagonal planes, or their effects in diagonal planes were not investigated. In this work, we aim to have low cross-polarization in all azimuth planes, including diagonal planes where cross-polarization is most challenging to suppress [22,23]. As our metric for cross-polarization is high, careful coordination of multiple techniques is required to meet the requirement because our preliminary studies showed that using a single method does not meet the design goals.

This paper proposes a novel high-performance dual-polarized antenna that can be used for many applications, such as remote sensing and satellite communication, where compact antennas with low cross-polarization are required. Alternatives are corrugated horns that are bulky, heavy, and very costly to fabricate. The key antenna parameters are determined to broaden the bandwidth of the antenna simultaneously for the impedance, high gain, high aperture efficiency, and low cross-polarization, which results in the most compact antenna size. Simultaneous improvement of multiple antenna performance parameters was achieved by carefully selecting and deriving a compatible set of performance enhancement techniques. The design considerations and the basic antenna configuration are presented in Section 2. Section 3 demonstrates several numerical studies on critical antenna elements/dimensions. To verify the simulation results, an antenna prototype was fabricated, and the test results are discussed in Section 4. Lastly, we present our conclusions and propose directions for future work in Section 5.

## 2. Design Concepts

**Figure 1:**
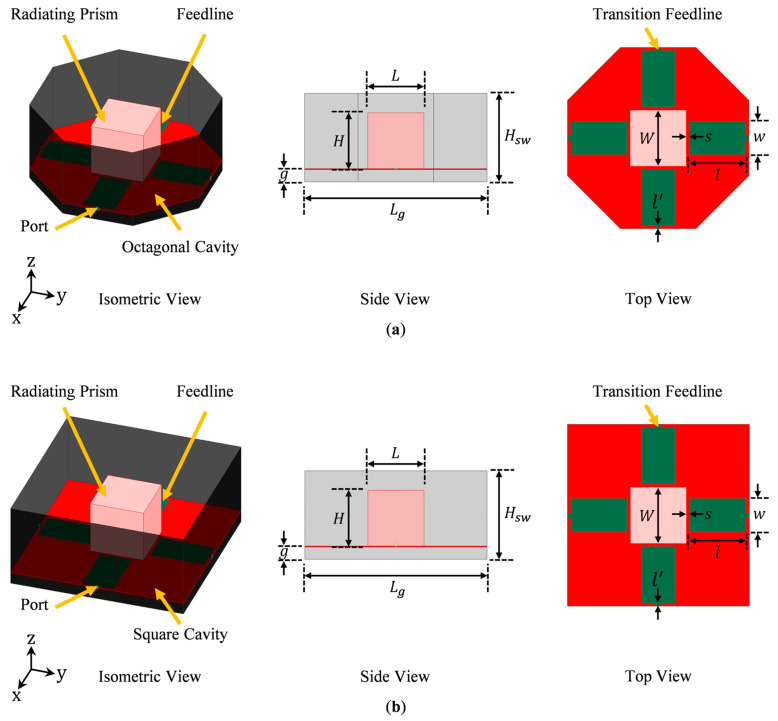
Geometries of cavity-backed radiating open prism antennas: (**a**) With an octagonal ground cavity; (**b**) With a conventional square ground cavity. (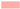 conductive hollow open prism with no bottom face), 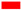 supporting dielectric substrate, 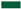 feedlines, 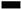 conductive ground cavity).

Our goal was to develop a novel dual-polarized antenna design that can provide wide impedance bandwidth, high gain with stable broadside radiation, low cross-polarization, and high aperture efficiency. Based on [17], we set the bandwidth (BW) and cross-polarization (X-pol) requirements to be ≥100 MHz and ≤−25 dB, respectively. Since there are multiple desirable qualities, careful coordination is required in order to simultaneously meet all of them. The designed antenna is shown in Figure 1a. The corners of its ground cavity are truncated to form an octagon shape. Figure 1b shows the case without the corner truncation. We will compare the merits of each configuration in Section 3.1.

### 2.1. Design Considerations for Antenna Components

A typical antenna design includes three major parts: ground, primary radiator, and feed. Based on the above-mentioned characteristics of interest, some of the basic design considerations are laid out below.

#### 2.1.1. Ground Structure

Due to its capacity for providing high gain with stable broadside radiation across a wide operating frequency band, a cavity-backed configuration was adopted for this design [13]. With a cavity-backed antenna, there is current on the ground cavity sidewalls. This can potentially extend the aperture field beyond the physical aperture of the antenna, consequently resulting in a bigger effective aperture area and a higher aperture efficiency than those of an antenna with a planar ground plane [24].

A basic ground cavity is formed by adding vertical sidewalls on all the edges of a conventional planar ground plane. Therefore, the shape of a ground cavity is dictated by that of the horizontal base of the cavity. One of the most frequently used planar ground planes is square in shape, and as a result, ground cavities with square [10,11] aperture openings are the most common. As shown in [16], compared to a square ground plane with sharp corners, a corner truncated planar ground plane allows lower cross-polarization. Since low cross-polarization is desirable for the applications of interest, a corner truncated ground plane cavity was adopted in this work.

#### 2.1.2. Primary Radiator

To achieve a wide impedance bandwidth, a hybrid design with multiple radiators was developed. To simplify the design, the feed structure was configured to serve as the extra radiator. Together with the primary radiator, they produce multiple adjacent resonances, which can be merged to form a wide bandwidth. The overall impedance bandwidth due to the resonance merging depends on the bandwidth of each individual radiator. Therefore, individual radiators having a wide bandwidth is advantageous to the overall bandwidth enhancement. To keep the design process simple, this work mainly focused on deriving a wideband primary radiator.

**Figure 2:**
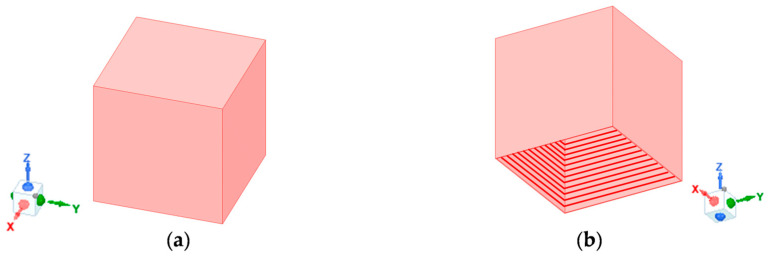
Open prism without a bottom face: (**a**) Trimetric view from above; (**b**) Trimetric view from below (inside of the open prism is shaded with red lines).

For a compact antenna, the primary radiator needs to be compact while simultaneously providing sufficiently long current paths on the feedlines, thereby permitting them to be efficient radiators. This can be achieved using a 3D radiator instead of a planar one. Due to its folded sides, a 3D radiator has a smaller footprint but supports longer current paths. Since the primary radiator is backed by a ground cavity with sidewalls, the use of a 3D radiator does not necessarily lead to a bigger overall antenna profile (i.e., it does not increase the overall height of the antenna). To support wideband or multiband operation, multiple current paths of different lengths should be allowed on the primary radiator [25]. This requires the 3D radiator to be sufficiently large along the Z-axis. Combining these design constraints, we proposed a hollow metallic open prism with no bottom face, as shown in Figure 2, to be the primary radiator. Dual-polarization was achieved by orthogonally exciting the open prism along the X- and Y-axes, i.e., the two orthogonal polarizations of interest. To ensure identical performance in the two polarizations and suppress cross-polarization, the open prism has an equal length along the X and Y-axes.

#### 2.1.3. Feeding Structure

Commonly used feeding structures include coaxial probes and printed feedlines. Both types of feeding structures can excite an antenna through direct contact or proximity coupling. With proximity coupling, the primary radiator is excited through the gap between it and the feeding structure. This allows a higher degree of freedom for tuning the impedance matching of the primary radiator and the resonant frequency of the feedline. Therefore, proximity coupling was adopted. Compared to a coaxial probe, a printed feedline has additional degrees of freedom to match an antenna, such as the shape and the width of the feedline. In addition, a printed feedline with a potentially larger width can provide stronger coupling than a coaxial probe with a limited diameter. Hence, in this work, we used printed lines as the feeding structure. To realize dual-polarized operation, the feedlines are placed orthogonally along the X- and Y-axes. To suppress cross-polarization, differential feeding was implemented. By adjusting the excitation/matching conditions of the orthogonal differential pairs, three dual-polarization schemes, namely dual-linear, slant dual-linear, and dual-circular polarizations, could be realized.

As established previously, in addition to its role in exciting the primary radiator, the feeding structure in this design needs to act as a broadside radiator at a close enough frequency to that of the primary radiator to allow resonance merging. The feedlines were suspended above the horizontal base of the ground cavity to provide broadside radiation. One end of each feedline is connected to an excitation port located in the ground cavity sidewall, while the other end is open to provide proximity coupling to the primary radiator (i.e., the radiating open prism). This way, the feedlines act as physically wide horizontal monopole antennas connected to a vertical ground plane and backed by a horizontal ground plane. Their large width makes them broadband capable. 

### 2.2. Antenna Configuration

Based on the above design considerations, we propose a novel design—a dual-polarized octagonal cavity-backed antenna with two orthogonal pairs of differential feedlines that excite a radiating open prism in the center of the cavity. We call this design an “octagonal cavity-backed open radiating prism,” or OCROP. Our antenna consists of four parts: (1) a conductive octagonal-shaped cavity, (2) a conductive radiating open prism, (3) two orthogonal pairs of differential feedlines, and (4) a supporting dielectric substrate. The geometry is shown in Figure 1a. The term “open prism” comes from the radiating element at the center of the cavity. The radiating element is a hollow metallic prism that is open at the bottom side. As an example, the case with a cubic prism essentially uses a hollow box with open bottom. The copper feedlines are placed on the top side of the supporting substrate. For high mechanical stability and low mass, the substrate should be a relatively thin woven dielectric with low permittivity. Here, we used a 0.762 mm thick Rogers CuClad 217 ($\varepsilon_{r}$ = 2.17 and tanδ = 0.0009). The initial value of the aperture length (*L_g_*) was 165 mm, the cavity height (*H_sw_*) was 80 mm, the gap between the lower dielectric layer and the ground plane (*g*) was 11 mm, and the spacing between the feedline and the radiating prism (*s*) was 3 mm. The feedline lengths and widths (*l* and *w*) were 53 mm and 30 mm, respectively. The transition feedline lengths and widths (*l’* and *w’*) were 1 mm and 3.6 mm, respectively. These values were selected to make the antenna operate in the L-band (1–2 GHz) with a resonance merging effect.

We considered numerous design configurations and performed parametric simulation studies to test the concepts. Since the antenna was designed for dual-polarized operation, the length and the width of the open prism needed to be the same, i.e., *L* = *W*, to allow geometrical symmetry and identical performance in two orthogonal polarizations of interest. For the antenna to operate in the L-band (1–2 GHz) around a center frequency of 1.5 GHz, the dimensions of the open prism were set to *L* = *W* = *H* = 51 mm, forming a cube with no bottom face (thus, the name “open” prism, as it has an open side). This value was found through simulation studies that are not included here for brevity.

## 3. Numerical Analysis

The numerical simulations throughout this study were performed using ANSYS Electronics Desktop 2021 R1©. Lumped port excitations with a constant full port impedance of 50 Ω were assigned to all four ports. In the following studies, only the Y-polarized differential pair was excited with equal amplitude and 180° out-of-phase signals, while the X-polarized differential pair was terminated with 50 Ω matched loads. Since the antenna is symmetric, X- and Y-polarizations have identical performance.

### 3.1. Benefits of an Octagonal Ground Cavity

**Figure 3:**
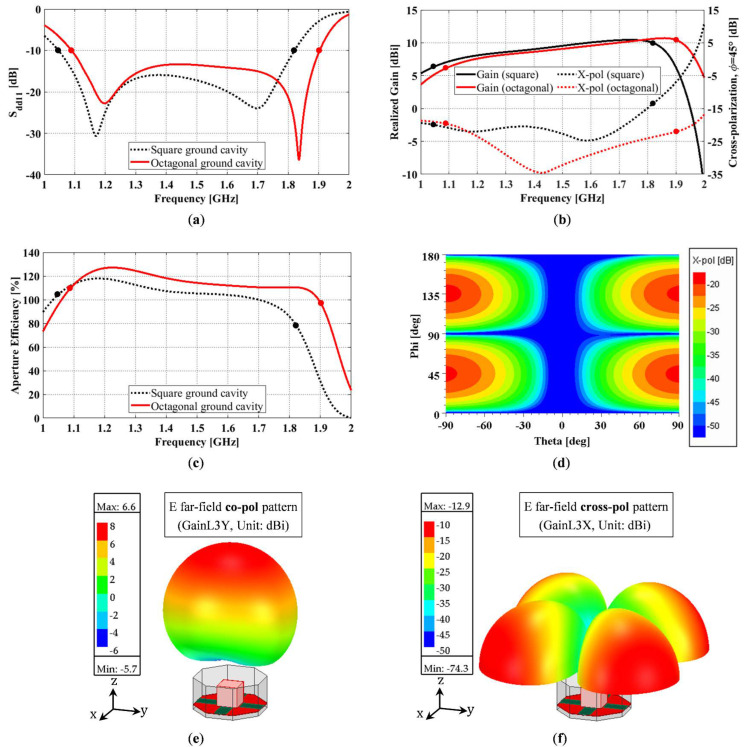
Square and octagonal ground cavity-backed radiating open prism antennas: (**a**) Differential reflection coefficient; (**b**) Realized gain and peak cross-polarization; (**c**) Aperture efficiency; (**d**) Cross-polarization ratio of OCROP for 1.1 GHz at all azimuth and elevation angles of interest; (**e**) 3D far-field co-polarized radiation pattern of the octagonal cavity-backed antenna at 1.1 GHz; (**f**) 3D far-field cross-polarized radiation pattern of the octagonal cavity-backed antenna at 1.1 GHz. (Black and red dots mark the lower and upper limits of the two S_dd11_ ≤ −10 dB operating bands.).

To verify the superiority of an octagonal ground cavity (Figure 1a) over a square one (Figure 1b), we compared the performance of an open prism antenna backed by these two types of ground cavities. All other antenna dimensions remained constant, with *L_g_* = 165 mm, *H_sw_* = 80 mm, *g* = 11 mm, *s* = 3 mm, *l* = 53 mm, *w* = 30 mm, *l’* = 1 mm, and *w’* = 3.6 mm. Figure 3a shows the differential reflection coefficient of the two antennas. The ground corner truncation leads to a small increase in the absolute impedance bandwidth (i.e., from 774 MHz to 813 MHz). With operating frequencies slightly shifting upward, the fractional impedance bandwidth does not change much. More specifically, both antennas offer a wide S_dd11_ ≤ −10 dB impedance bandwidth: the one with the square ground cavity has an impedance bandwidth of 54.0%, and the one with the octagonal ground cavity has an impedance bandwidth of 54.3%. The gain and diagonal-plane cross-polarization of the two antennas are compared in Figure 3b. The average gains across the corresponding impedance bandwidths of the square and the octagonal ground cavity-backed antennas are almost the same, with the former being 9.1 dB and the latter being 9.0 dB. By contrast, the effect of the ground corner truncation on cross-polarization is more evident. It is worth clarifying that the cross-polarization values in negative dB refer to peak cross-polarization (ratio), i.e., the difference between the peak cross-polarization (in dBi) and the peak co-polarization (in dBi) in the specified ϕ-plane (in this case, ϕ = 45°) within the entire θ range of interest (i.e., −90° ≤ θ ≤ 90°), not cross-polarization on the axis (i.e., θ = 0°). With the square ground cavity, across the operating frequency band, the average peak diagonal-plane cross-polarization is −21.2 dB, and the cross-polarization requirement of ≤ −25 dB is not met at any frequencies; with the octagonal ground cavity, the average peak diagonal-plane cross-polarization is −27.3 dB across the operating frequency band, and 65.4% of the operating frequencies meet the cross-polarization requirement. This verifies that the use of a corner truncated cavity can effectively suppress the cross-polarization in the diagonal planes. In addition to the above-mentioned advantage, the antenna with the octagonal ground cavity also enjoys a smaller (by 17.2%) footprint and a higher (by 9.3%) average aperture efficiency, as shown in Figure 3c. More precisely, the average aperture efficiencies across the corresponding operating frequency bands of the square and the octagonal cavity-backed antennas are 106.4% and 115.7%, respectively. Figure 3d shows the cross-polarization ratio of the octagonal cavity-backed antenna, at 1.1 GHz, in all elevation and azimuth planes. It verifies that: the maximum cross-polarization of the proposed antenna occurs in the diagonal planes (i.e., ϕ = 45° and 135°), and there is zero cross-polarization in the principal planes (i.e., ϕ = 0° and 90°). Therefore, we can use the peak diagonal plane cross-polarization to represent the maximum cross-polarization in all ϕ planes (0° ≤ ϕ ≤ 360°). The frequency of 1.1 GHz was chosen because it has the worst cross-polarization performance across the operating frequency band. Figure 3e,f shows the 3D far-field co- and cross-polarized radiation of the octagonal cavity-backed antenna at 1.1 GHz. They were computed using the predefined HFSS functions, i.e., GainL3Y and GainL3X, that are based on Ludwig’s 3rd definition [26]. The overlay co-polarized field shows that the antenna radiates in its broadside. The overlay cross-polarized field verifies the same information as Figure 3d, i.e., cross-polarization does not exist in the principal planes and reaches its maximum in the diagonal planes.

### 3.2. Realization of Wide Impedance Bandwidth

Our design attains wide impedance bandwidth by using multiple radiators that have adjacent resonances and similar radiation patterns (in our case, broadside radiation patterns). These radiators are the open prism and the feedlines. 

**Figure 4:**
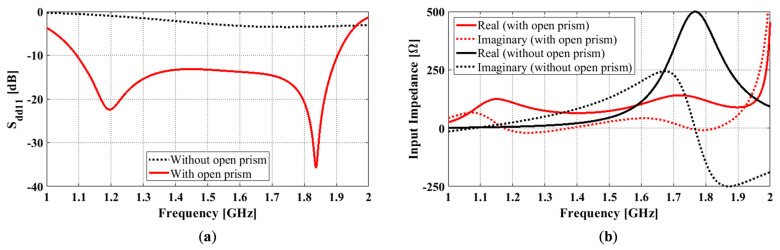
The octagonal cavity-backed antennas with and without the open prism: (**a**) Differential reflection coefficient; (**b**) Differential input impedance.

To demonstrate the resonance merging mechanism of this antenna, the differential reflection coefficients and input impedances of the antennas with and without the open prism are compared in Figure 4a,b. All other antenna dimensions remained constant, with *L_g_* = 165 mm, *H_sw_* = 80 mm, *g* = 11 mm, *s* = 3 mm, *l* = 53 mm, *w* = 30 mm, *l’* = 1 mm, and *w’* = 3.6 mm. As can be seen, the differential reflection coefficient for the antenna without the open prism (black dotted curve in Figure 4a) only has one notch, which is not very pronounced due to the antenna being poorly matched. However, this can be verified with the peak in the real part of the corresponding input impedance curve (black solid curve in Figure 4b). By adding the open prism, the antenna becomes well-matched. An extra notch in the reflection coefficient curve is introduced (red solid curve Figure 4a), which is also reflected by the extra bump in the real part of the input impedance curve (red solid curve Figure 4b). As the two notches are close together, they result in a merging effect, and a wide impedance bandwidth of 54.3% is created.

### 3.3. Effect of Air Gap

Among all the antenna dimensions, except for the air gap, g, and cavity sidewall height, *H_sw_*, the rest are directly related to the physical dimensions of the two radiators. Although the air gap does not affect the physical dimensions of any antenna components, it determines the spacing between the ground plane and the open prism/feedlines. We performed a parametric study on the air gap to understand its effects on the antenna performance. In our studies, the air gap varied from 10 mm to 16 mm with a 1 mm increment. All other antenna dimensions remained constant, with *L_g_* = 165 mm, *H_sw_* = 80 mm, *s* = 3 mm, *l* = 53 mm, *w* = 30 mm, *l’* = 1 mm, and *w’* = 3.6 mm.

**Figure 5:**
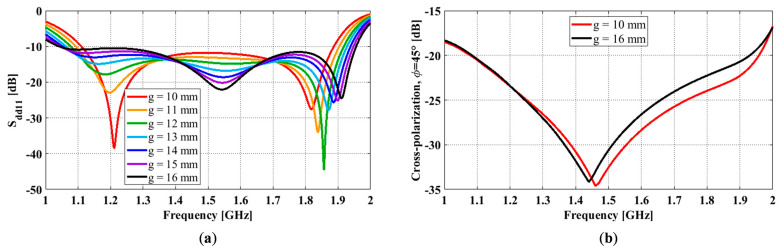
OCROP with different air gaps: (**a**) Differential reflection coefficient; (**b**) Peak cross-polarization ratio.

The differential reflection coefficient results for different air gaps are given in Figure 5a. It shows that the lower frequency shifts down and the upper frequency shifts up as the air gap increases from 10 mm to 16 mm, which leads to an 8.8% increase in the impedance bandwidth (from 51.7% to 60.5%). However, a further increase in the air gap may raise certain sections of the reflection coefficient (i.e., around 1.2 GHz and 1.8 GHz) above −10 dB and lead to a smaller impedance bandwidth. By observing the real part of their input impedances, we can see that the number of peaks in the real part of these curves within the operating frequency bands remains the same (i.e., 2) for all cases. Figure 5b shows the peak diagonal plane cross-polarization of the antennas with different air gaps. Here, we only show the cases g = 10 mm and g = 16 mm because the rest follow the same trend. As can be seen, the effect of the air gap on the cross-polarization is negligible at frequencies ≤ 1.45 GHz. Above 1.45 GHz, an increase in the air gap leads to a slightly higher cross-polarization ratio. Thus, a smaller cross-polarization bandwidth. In this paper, the cross-polarization bandwidth is a measure of the frequency range that meets the design requirement of X-pol ≤ −25 dB.

As can be seen, at this stage, the impedance bandwidth (i.e., for S_dd11_ ≤ −10 dB) is much wider than the cross-polarization bandwidth (i.e., for X-pol ≤ −25 dB). For instance, the -impedance bandwidth for the case g = 11 mm is 54.3%, while its cross-polarization bandwidth is 34.9%.

### 3.4. Effect of Ground Cavity Sidewall Height

For a cavity-backed antenna, its radiation performance is heavily dependent on the ground cavity [13]. Two key parameters of a ground cavity are its aperture size (*L_g_*) and cavity height (*H_sw_*). Since the excitation ports of the antenna are located on the cavity sidewalls, the feedline length is directly correlated with the aperture length. In other words, the impedance bandwidth is very sensitive to changes in the aperture length. To improve the radiation performance with minimal impact on its impedance bandwidth, here, we only investigate the effect of the ground cavity sidewall height. To determine this, we varied the sidewall height *H_sw_* from 70 mm to 140 mm in 10 mm increments while keeping all other antenna dimensions constant (i.e., *L_g_* = 165 mm, *g* = 11 mm, *s* = 3 mm, *l* = 53 mm, *w* = 30 mm, *l’* = 1 mm, and *w’* = 3.6 mm).

**Figure 6:**
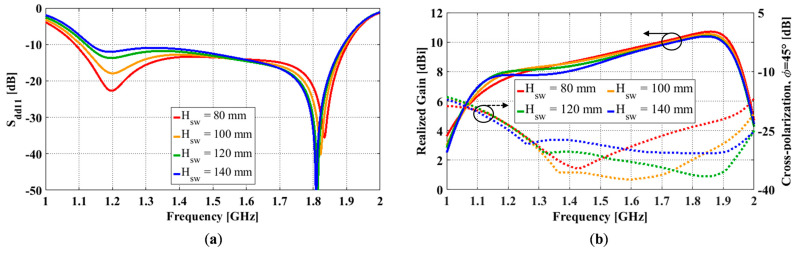
OCROP with different sidewall heights: (**a**) Differential reflection coefficient; (**b**) Realized gain (solid, left y-axis) and peak cross-polarization ratio (dotted, right y-axis).

Simulated differential reflection coefficient (S_dd11_) results for different sidewall heights are shown in Figure 6a. Lower and upper frequencies for S_dd11_ ≤ −10 dB are $f_{1}$ and $f_{2}$. A center frequency, $f_{c}$, was calculated as the mean value of $f_{1}$ and $f_{2}$ and these values were used to calculate the impedance bandwidth. For all sidewall heights, there was a notch at 1.2 GHz and approximately 1.83 GHz. As the height was increased from 70 mm to 140 mm, the notch at 1.2 GHz increased, and the notch at 1.83 GHz deepened. Simultaneously, the impedance bandwidth slightly decreased from 55.2% to 50.0%. Sidewall heights around 80 mm showed a good balance between the two notch depths, and the curve remained below −10 dB.

The effect of varying the sidewall height on the gain and the diagonal plane (ϕ = 45°) cross-polarization ratio is shown in Figure 6b. The gain at the low end of the impedance bandwidth ($f_{1}$) is around 6.5 dBi and increases to a peak of 10.7 dBi at the upper frequency ($f_{2}$). There are small variations in gains in relation to different sidewall heights, and the trend is relatively consistent between the simulation results. As the sidewall height increases from 70 mm to 140 mm, the cross-polarization bandwidth significantly increases from 25.8% to 49.2%. Varying the sidewall height had little effect on the cross-polarization at the lower end of the operating band but significantly affected the cross-polarization in the mid and upper sections of the operating band. The minimum cross-polarization at $f_{1}$ occurs when the sidewall height reaches 100 mm. Beyond this height, the cross-polarization ratio rises in the mid-section of the operating band. The minimum cross-polarization at $f_{2}$ occurs when the sidewall height reaches 120 mm.

The bandwidth of operation is summarized in Table 1. As the sidewall height increases, impedance bandwidth (for S_dd11_ ≤ −10 dB) decreases while cross-polarization bandwidth (for X-pol ≤ −25 dB) increases. In column 4, we present the combined bandwidth, where we took the minimum and maximum frequencies that met both requirements. In all cases, the lower frequency bound, $f_{1}$, is limited by the cross-polarization ratio. The upper frequency bound, $f_{2}$, is limited by the impedance bandwidth for *H_sw_* ≤ 90 mm and the cross-polarization bandwidth for *H_sw_* ≥ 100 mm. Once the sidewall height reaches 90 mm, there is very little change in the overall operating bandwidth (42.7 ± 0.7%).

**Figure 7:**
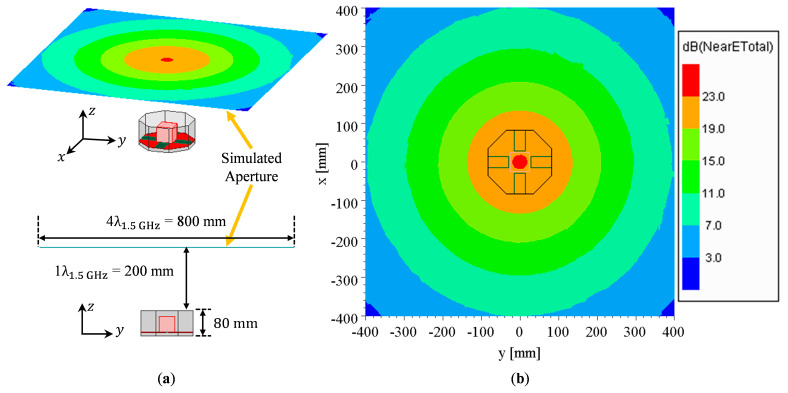
OCROP near field distribution for 1. 5 GHz at 1λ above the aperture opening: (**a**) Oblique view; (**b**) Bottom view.

The aperture field distribution for the antenna, with *g* = 11 mm and *H_sw_* = 80 mm, at 1.5 GHz on a fictitious square surface with a length of 4 λ, located 1 λ above the aperture, as shown in Figure 7 exhibits a uniform characteristic, with large peak electric field magnitudes immediately above the antenna. As observed from the figure, the aperture field extends well beyond the physical aperture of the antenna, which causes the gain enhancement and aperture efficiency to be greater than 100%.

## 4. Antenna Prototype Fabrication and Test Results

In this section, we present measurement results from a prototype of the OCROP antenna. We start by presenting the feeding network, which was formed with a 180° hybrid coupler. Next, we present the reflection coefficient and radiation pattern measurements for the prototype antenna to verify their performance with respect to the simulations.

In practice, we require a differential feed when using the antenna and measuring radiation patterns. Therefore, two types of measurements are required.

First, we needed to establish the differential S-parameters to compare the simulated and fabricated designs. The two-port VNA in our lab only supports single-ended S-parameter measurements. Since two of the ports are fed simultaneously, we cannot simply use the standard S-parameter measurement approach (which assumes a matched load at all other ports except for the measured port). Additionally, we cannot measure the differential reflection coefficients in our lab using the two-port VNA. Therefore, to characterize the differential feed, we measured the single-ended S-parameters at all ports (with all other ports match terminated) and calculated the differential reflection coefficients using an established approach [27].

Secondly, to use the antenna in practice, we need to provide a single input and a feed network. This is required in our facility to measure the radiation pattern, as the receiver has a single port for measurement. Therefore, we measured the input reflection coefficient, as seen at the input to the combination of the feeding network and antenna together. These results are presented in this section.

### 4.1. Differential Feed Network

**Figure 8:**
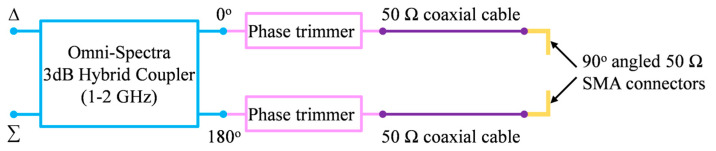
Schematic configuration of the differential feed network.

Ideal differential feeding requires equal amplitude (i.e., 0 dB amplitude imbalance) and 180° phase difference at the two differential ports. This was easily realized in HFSS simulation by setting up the correct amplitude/phase for each excitation port and assigning differential pairs. In measurement, a feed network (shown in Figure 8) was constructed from an L-band (i.e., 1–2 GHz) 3 dB 180° hybrid coupler, phase trimmers, 50 Ω coaxial cables, and 90° angled SMA adaptors.

Since the antenna ports are in the sidewalls of the ground cavity, we used 90° angled SMA connectors to eliminate the stress of the cable mass on the prototype, and these were used to connect the antenna to the feed network. Without the phase trimmers, the phase difference between the signals from port 0° and port 180° of the coupler and cable combination exhibits relatively large deviations from the ideal value of 180°.

A vector network analyzer (i.e., VNA) was used to evaluate the S-parameters of the feed network and the antennas. The magnitude imbalance at the end of the cables was ≤0.5 dB within the 1.05 to 2.0 GHz bandwidth. After adding the phase trimmers, the differential phase error within 1.05 to 1.9 GHz bandwidth was ≤5°. So, in measurement, the feed network with the phase trimmers was used to provide differential signals to both fabricated antennas.

### 4.2. OCROP Antenna Prototype

**Figure 9:**
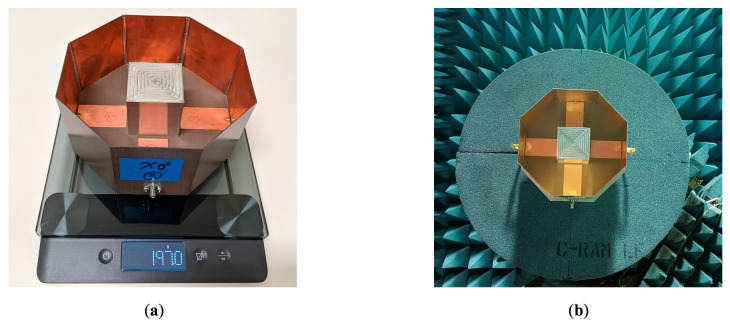
OCROP prototype: (**a**) Situated on mass scale; (**b**) In the test chamber for radiation patterns.

A prototype of the differentially-fed, dual-polarized OCROP antenna, as shown in Figure 9, was fabricated and tested. Printed circuit board (PCB) substrates with copper on one side were used for constructing the ground cavity, as this had the dual benefit of being easy to solder and having low mass (compared to a solid piece of the copper plate). Since the dielectric material of the PCB substrate was behind the copper ground cavity, its material properties had minimal effect on the antenna performance. Due to the material available at the fabrication facility, DiClad 527 ($\varepsilon_{r}$ = 2.5 and  tanδ = 0.0018) with a thickness of 0.762 mm was used. As described earlier, the open prism is a 51 mm × 51 mm × 51 mm bottomless hollow cube. The supporting substrate for the feedlines and the open prism is 0.762 mm thick Rogers CuClad 217. For better alignment and easier assembly of the prism, a 51 mm × 51 mm relief was routered in the center of the substrate. The depth of the relief is half the substrate thickness. The open prism was then placed in the square relief. The open prism was milled from an aluminum cube and had a wall thickness of 0.4 mm. The supporting substrate was suspended 15 mm above the ground plane. i.e., g = 15 mm. The prism was mechanically attached to the supporting substrate with a small stainless-steel screw in each bottom corner. The ground plane length and sidewall height were 165 mm and 80 mm, respectively. Detailed antenna dimensions are given in Table 2. The reference dimensions can be seen in Figure 1.

**Figure 10:**
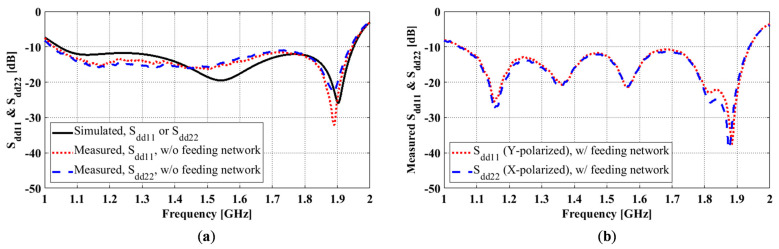
OCROP differential reflection coefficient simulation and measurement: (**a**) Without feeding network; (**b**) With feeding network.

To evaluate the differential reflection coefficients of the antenna, we conducted two types of measurements with a two-port VNA. The first type measured single-ended S-parameters at all antenna ports. The differential reflection coefficients were obtained from the post-processing of these data by assuming differential signals for one differential pair and matched loads for the other pair. In simulations, the reflection coefficients were calculated using the same post-processing approach (an already established function in HFSS) but with the single-ended S-parameters obtained numerically in HFSS. Both simulated and measured differential reflection coefficients using this method are shown in Figure 10a. S_dd11_ and S_dd22_ are Y- and X-polarized differential reflection coefficients, respectively. As can be seen, there is good agreement between the simulated and measured results in terms of the operating frequency range where S_dd11_ and S_dd22_ ≤ −10 dB.

To provide differential feeding to the antenna, we used the feed network described in Section 4.1. The 3 dB 180° hybrid coupler divides the input signal from the differential port (△) into two output signals with equal amplitude and opposite phases at port 0° and port 180° of the coupler. In the differential reflection coefficient measurement, the port △ of the coupler was connected to the VNA, and the coupler ports 0° and 180° were connected to the antenna ports 0° and 180°. The measured results showed a 0.5 dB magnitude imbalance and 176.6° phase difference at the two differential ports for 1.5 GHz.

Figure 10b shows the reflection coefficient measured at the input to the combined feeding network and antenna. Several additional notches appeared in the measurement, which was attributed to the effects of the hybrid coupler. As can be seen, the impedance bandwidth of the operation is the same as the simulation. The measured S_dd11_ and S_dd22_ would be slightly different if a different feeding network were to be used, and therefore, in practice, the feeding network must be carefully selected and designed.

**Figure 11:**
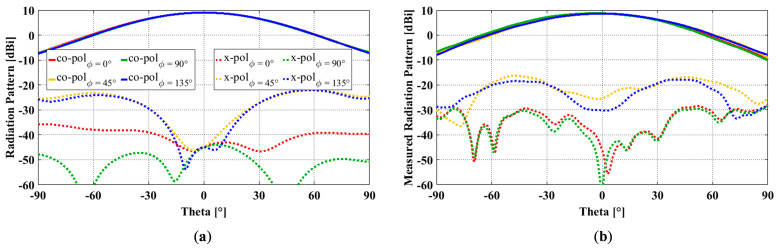
OCROP radiation patterns at 1.5 GHz: (**a**) Simulated results; (**b**) Measured results.

Radiation patterns were measured in a Compact Antenna Test Range. The simulated and measured results of the fabricated antenna are compared in Figure 11. Figure 11a,b compare the simulated and measured radiation patterns at 1.5 GHz in different ϕ-cut planes, namely principal planes of ϕ = 0° and 90° and diagonal planes of ϕ = 45° and 135°. The measured gain (8.6 dBi), accounting for the insertion loss from the feed network, is 0.4 dB lower than the simulated gain (9.0 dBi). Compared to the simulated X-pols, the measured ones are higher in all four planes. More specifically, the measured X-pols in the ϕ = 0°, 45°, 90°, and 135° planes are −36.5 dB, −25.0 dB, −37.0 dB, and −26.5 dB, respectively, while the simulated X-pols accounting for the amplitude and phase errors are −44.8 dB, −31.2 dB, −53.0 dB, and −31.1 dB in the four corresponding planes, respectively.

In the measurement, there are two sources of errors for cross-polarization, i.e., the Compact Range reflector and the antenna mount. The Compact Range reflector is an offset reflector, and its cross-polarization is limited to −35 dB. The scattering from the antenna mount on the tower and the tower itself contributes to the cross-polarization. Despite the differences, the measured X-pols at 1.5 GHz still met the X-pol requirement of ≤ −25 dB in both principal and diagonal planes. In addition to the excellent electrical performance, the fabricated antenna is also lightweight (197.0 g) and compact (0.825 λ × 0.825 λ × 0.4 λ at the center frequency of 1.5 GHz).

## 5. Conclusions

In this paper, the design procedure of a high-performance octagonal cavity-backed antenna was presented. The proposed antenna exhibits multiple attractive qualities: dual-polarization, wide impedance bandwidth, low cross-polarization, high gain, and high aperture efficiency. It can be used for many applications, such as remote sensing and satellite communication, where compact antennas with low cross-polarization are required. Alternatives are corrugated horns that are bulky, heavy, and very costly to fabricate. The cavity-backed configuration ensures that the antenna supports stable broadside radiation with relatively high gain and low cross-polarization. An octagonal ground cavity, instead of a more commonly seen square one, was used because it offers lower cross-polarization and higher aperture efficiency while being more compact. By merging the resonances from the open prism and the radiating feedlines, wide impedance bandwidth was realized. The bandwidth can reach over 60% by adjusting the air gap between the two radiators and the ground plane. Multiple design considerations were adopted to ensure low cross-polarization. Some basic ones include ensuring a symmetric antenna configuration, adopting a differential feed, and feeding the antenna from the ground sidewalls instead of the bottom ground plane. Further cross-polarization reduction can be realized by moderately increasing the cavity sidewall height. To verify the design and fabrication process, an antenna prototype was fabricated and tested with excellent agreement between the simulated and measured results. 

To demonstrate the superior performance of the proposed antenna, we compared it with three high-performance cavity-backed antennas with wide impedance bandwidth in Table 3. Unfortunately, the majority of the antennas in the literature only aim to suppress cross-polarization in the principal planes (i.e., P-planes) and do not provide information on cross-polarization in the diagonal planes (i.e., D-planes) where it peaks, e.g., [13,28]. As can be seen, compared to these antennas, our antenna is compact and has the widest impedance bandwidth, lowest peak (across the entire impedance bandwidth), diagonal-plane cross-polarization, and highest aperture efficiency.

It has shown that there is a small section of the operating band (i.e., lower frequencies) that is not sensitive to the cross-polarization suppression techniques used here. We will propose an additional cross-polarization suppression technique to overcome this issue in the future. In addition, since some applications require higher gain than reported here, we will investigate different gain enhancement techniques that are compatible with our design.

## Figures and Tables

**Table 1:** OCROP antenna bandwidths (BWs) and gain as a function of sidewall height.

H_sw_ (mm)	Impedance BW (S_dd11_ ≤ −10 dB) (%)	X-pol BW (Peak ≤ −25 dB) (%)	Combined BW (%)	Av. Gain (Across Combined BW) (dBi)
70	55.2	25.8	25.8	8.70
80	54.3	34.9	34.9	9.05
90	53.6	42.0	42.0	9.34
100	52.6	45.3	42.6	9.23
110	51.8	46.7	42.4	9.22
120	51.1	47.7	42.4	9.18
130	50.4	48.4	43.0	8.99
140	50.0	49.2	43.9	8.97

**Table 2:** OCROP prototype dimensions (all dimensions in mm).

*L* = *W* = *H*	*L_g_*	*H_sw_*	*g*	*s*	*l*	*w*	*l’*	*w’*
51	165	80	15	3	53	30	1	3.6

**Table 3:** Performance comparison of the proposed antenna with similar antennas.

Antenna Type	Polarization	Aperture Area	Antenna Height	Imp. BW (%)	Peak Gain across Imp. BW (dBi)	Peak D-plane X-pol across Imp. BW (dB)	Aperture Efficiency @ f_c_ (%)
Waveguide fed short backfire [29]	Single	$4.155\;\lambda_{c}{}^{2}$	0.732 λ_c_	43.5	15.8	≥0	72.7
Composite cavity backed bowtie [13]	Dual	$0.830\;\lambda_{c}{}^{2}$	0.257 λ_c_	39.2	10.7	-	88.7
Square cavity backed bowtie [28]	Dual	$0.538\;\lambda_{c}{}^{2}$	0.293 λ_c_	44.5	8.6	-	89.0
This antenna	Dual	$0.561\;\lambda_{c}{}^{2}$	0.399 λ_c_	54.3	10.7	≤−20	114.2

## Data Availability

Data available within the article.
